# Death of Dr. Lucien B. Johnson

**Published:** 1889-04

**Authors:** 


### DEATH OF DR. LUCIEN B. JOHNSON.

The profession of Texas, and especially of Austin, has suffered a sad loss in the death of Dr. Johnson. He was a native of Williamsburg, Ohio, but removed South many years ago, and in 1880 came to Travis county, Texas. He located at Del Valle, near Austin, and engaged in farming in connection with the practice for awhile, and removed to Austin in 1882.

Dr. Johnson was exceedingly popular; being benevolent, kind-hearted and hospitable, he won all hearts, and his death is mourned by all classes in Austin. He was a member of the Austin District, and the Travis County Medical Societies, and also of the Physicians' Mutual Benefit Association. He was also a Knight Templar, and his funeral, which occurred April 11, and was largely attended, was conducted under the auspices of that grand Order,—his confreres of the medical profession acting as pall-bearers. The ceremonies were most touching and impressive, and were rendered more so by the funeral rites of the Episcopal Church, conducted by Rev. T. B. Lee. The coffin, and later the grave, was profusely covered with lovely flowers, tenderly laid there as a last tribute to the worth of a dear, departed friend.

